# Peripheral Organs of Dengue Fatal Cases Present Strong Pro-Inflammatory Response with Participation of IFN-Gamma-, TNF-Alpha- and RANTES-Producing Cells

**DOI:** 10.1371/journal.pone.0168973

**Published:** 2016-12-22

**Authors:** Tiago F. Póvoa, Edson R. A. Oliveira, Carlos. A. Basílio-de-Oliveira, Gerard J. Nuovo, Vera L. A. Chagas, Natália G. Salomão, Ester M. Mota, Marciano V. Paes

**Affiliations:** 1 Laboratory of Biotechnology and Physiology of Viral Infections, Oswaldo Cruz Institute, Oswaldo Cruz Foundation, Rio de Janeiro, Brazil; 2 Laboratory of Molecular Modeling, Federal University of Rio de Janeiro, Rio de Janeiro, Brazil; 3 Pathological Anatomy, Gaffrée Guinle Hospital, University of Rio de Janeiro, Rio de Janeiro, Brazil; 4 Ohio State University Comprehensive Cancer Center, Columbus, Ohio, United States of America; 5 Phylogeny Inc, Powell, Ohio, United States of America; 6 Pathological Anatomy, Clementino Fraga Filho University Hospital, Rio de Janeiro, Brazil; 7 Interdisciplinary Laboratory of Medical Research, Oswaldo Cruz Institute, Oswaldo Cruz Foundation, Rio de Janeiro, Brazil; 8 Laboratory of Pathology, Oswaldo Cruz Institute, Oswaldo Cruz Foundation, Rio de Janeiro, Brazil; University of Hong Kong, HONG KONG

## Abstract

Dengue disease is an acute viral illness caused by dengue virus (DENV) that can progress to hemorrhagic stages leading to about 20000 deaths every year worldwide. Despite many clinical investigations regarding dengue, the immunopathogenic process by which infected patients evolve to the severe forms is not fully understood. Apart from differences in virulence and the antibody cross reactivity that can potentially augment virus replication, imbalanced cellular immunity is also seen as a major concern in the establishment of severe dengue. In this context, the investigation of cellular immunity and its products in dengue fatal cases may provide valuable data to help revealing dengue immunopathogenesis. Here, based in four dengue fatal cases infected by the serotype 3 in Brazil, different peripheral organs (livers, lungs and kidneys) were studied to evaluate the presence of cell infiltrates and the patterns of local cytokine response. The overall scenario of the studied cases revealed a considerable systemic involvement of infection with mononuclear cells targeted to all of the evaluated organs, as measured by immunohistochemistry (IHC). Quantification of cytokine-expressing cells in peripheral tissues was also performed to characterize the ongoing inflammatory process by the severe stage of the disease. Increased levels of IFN-*γ*- and TNF-*α*-expressing cells in liver, lung and kidney samples of post-mortem subjects evidenced a strong pro-inflammatory induction in these tissues. The presence of increased RANTES-producing cell numbers in all analyzed organs suggested a possible link between the clinical status and altered vascular permeability. Co-staining of DENV RNA and IFN-*γ* or TNF-*α* using *in situ* hibridization and IHC confirmed the virus-specific trigger of the pro-inflammatory response. Taken together, this work provided additional evidences that corroborated with the traditional theories regarding the “cytokine storm” and the occurrence of uneven cellular immunity in response to DENV as major reasons for progress to severe disease.

## Introduction

Dengue is considered the most important mosquito-borne viral disease due to its clinical relevance and rapid spread, nowadays putting at risk about half of the world’s population [1]. The etiologic agent, dengue virus (DENV), is distributed as four distinct serotypes (DENV1 to DENV4) and infections can result in a mild flu-like acute illness known as dengue fever (DF) [2]. From an epidemiological view, it is estimated that 390 million dengue infections occur each year, of which nearly 25% are symptomatic [3]. While most patients naturally recover from the non-severe clinical DF course, a small proportion evolves to severe disease, mostly characterized by plasma leakage and hemorrhagic manifestations (namely dengue shock syndrome—DSS and dengue hemorrhagic fever—DHF) [2, 4]. Despite the relevant mortality rates derived from dengue complications (arround 20000 deaths each year) [5], the elucidation of the pathogenic process by which infected patients evolve to the severe forms is still an ongoing challenge. Apart from the relationship between social determinants of health and dengue fatal cases, biological factors such as distinct virulence levels among virus strains and host immunity have been considered as key elements to drive patients to severe stages [6, 7]. Disease complications triggered by DENV enhanced infections assisted by previously-formed opsonizing antibodies, were related to altered T cell activation and cytokine production in secondary infections [8–10]. Yet, concerning a host primary response environment, other unknown factors could also play a role in triggering severe dengue. Classical DF symptoms, such as fever and headaches, usually match with high viremia levels, but interestingly the severe forms of dengue (DSS/DHF), when manifested, occur after virus clearance. This observation has raised concerns about the association of severe dengue with immonopathological mechanisms [11, 12].

In this context, the investigation of post-mortem severe dengue cases may represent a valuable tool for a better understanding of the immune scenario during a terminal stage. Additionally, a search for evidences regarding cell migration and cytokine production in peripheral tissues may also provide new insights about possible underpinning immune mechanisms linked to the development of severe forms. In a previous report of our laboratory, peripheral organs such as livers, lungs and kidneys of four dengue cases that died from DENV-3 were histopathologically and ultrastructurally screened [13]. Aside from virus detection in unusual sites such as hepatocytes and type II pneumocytes, all studied organs presented lesions that corresponded to severe dengue cases. In this work, the same post-mortem samples were object of study for investigation of the cellular immune response and its products. Immunohistochemical analysis revealed a systemic involvement of infection with mononuclear cells targeted to all of the analyzed tissues. Assessment of local cytokine response showed increased levels of IFN-*γ*- and TNF-*α*-expressing cells in livers, lungs and kidneys that evidenced a consistent pro-inflammatory induction in these tissues. Co-expression of DENV RNA and IFN-*γ* or TNF-*α* by Kupffer cells confirmed the specific DENV induction over the cytokine production, as found by *in situ* hibridization and IHC. Furthermore, an indicative of altered vascular permeability found in all analyzed organs was also suggested due to the presence of increased levels of local RANTES-producing cells.

Ultimately, this work brought additional evidences that the effect of the uneven cellular immunity in response to DENV can contribute to disease severity. Given the limited numbers of reports concerning investigation of post-mortem samples from dengue severe cases, this work importantly contributes to narrowing the gaps of dengue immunopathogenesis.

## Materials and Methods

### Ethical procedures

All procedures performed during this work were approved by the Ethics Committee of the Oswaldo Cruz Foundation/FIOCRUZ, with the number CAEE: 47525115.3.0000.5248 for studies with dengue fatal cases and controls. The use of tissue samples informed consent was verbally provided by family members to the responsible physician Dr. Carlos Alberto Basíllio de Oliveira by the time of the necropsy. This consent procedure was approved by the ethics committee.

### Human fatal cases

The human tissues analyzed in this study (livers, lungs and kidneys) were obtained from four dengue fatal cases that occurred during a Brazilian outbreak of DENV-3 in 2002 in Rio de Janeiro. All patients died with a clinical diagnosis of severe dengue with infections confirmed by positive serum IgM antibodies. The four negative controls, from both sexes and ranging from 40 to 60 year old, were non-dengue and did not present any other infectious disease. More information about these cases can be found in a previous report of our laboratory [13]. Briefly:

#### Case 1

A 63-year-old male patient that developed a sudden onset of headache, myalgia, anorexia and abdominal pain. A few days later the patient presented diarrhea, thrombocytopenia (platelet 79.000/mm^3^) and hemoconcentration (hematocrit 59%). The case eventually evolved to shock with severe pulmonary congestion followed by death with a clinical diagnosis of dengue hemorrhagic fever.

#### Case 2

A 21-year-old female patient who experienced fever, myalgia and headache with progression to metrorrhagia, nausea, vomiting and diarrhea. The patient also presented severe leukopenia and thrombocytopenia (platelet 10.000/mm^3^). During hospitalization, the case progressed to respiratory failure, followed by evolution of multiple organ failure and refractory shock.

#### Case 3

A 41-year-old female presenting fever, weakness, abdominal pain, leukocytosis, hematocrit of 48% and fluid in the abdominal cavity. The patient was diagnosed with dengue hemorrhagic fever and died from an acute pulmonary edema.

#### Case 4

A 61-year-old female that manifested classical dengue symptoms (fever, myalgia, vomiting and diarrhea). The patient evolved to severe clinics and died from acute pulmonary edema with sudden cardiac arrest.

### Histopathological analysis

Tissue samples from the human necropsies were fixed in formalin (10%), blocked in paraffin resin, cut in 4 *μ*m, deparaffinized in xylene and rehydrated with alcohol, as described previously [14]. Sections were stained with hematoxylin and eosin (H.E.) for histological examination and visualized under a Nikon ECLIPSE E600 microscope.

### Immunohistochemical procedure

For immunohistochemical studies, the paraffin-embedded tissues were cut (sections of 4 *μ*m), deparaffinized in xylene and rehydrated with alcohol. Antigen retrieval was performed by heating the tissue in the presence of citrate buffer [15]. Tissues were blocked for endogenous peroxidase with 3% hydrogen peroxidase in methanol and rinsed in Tris-HCl (pH 7.4). To reduce non-specific binding, sections were incubated for 30 min at room temperature. Samples were then incubated over-night at 4°C with anti-human antibodies that recognize CD4 (Spring Bioscence, CA, USA), CD8 (DAKOCytomation, CA, USA), CD20 (Biocare Medical, CA, USA), CD68 (Biocare Medical, CA, USA), IFN*γ* (Abbiotec, CA, USA), TNF*α* (Abbiotec, CA, USA), IL-10 (Abbiotec, CA, USA), RANTES/CCL5 (Santa Cruz Biotechnology, CA, USA) or TGF-*β* (Abbiotec, CA, USA). The next day, sections were incubated with rabbit anti-mouse IgG, a secondary antibody horseradish peroxidase (HRP) conjugate (Spring Bioscience, CA, USA), for 30 min at room temperature. For negative control of the immunohistochemical reactions, samples were incubated only with the secondary HRP-conjugated antibody. Reactions were revealed with diaminobenzidine (Dako, CA, USA) as a chromogen and the sections were counterstained in Meyer’s hematoxylin (Dako). Finally, samples were examined under a Nikon ECLIPSE E600 microscope.

### Quantification of positive cells by immunohistochemistry

Slides were evaluated using a Nikon ECLIPSE E600 microscope with a coupled Cool SNAP-Procf Color camera. For each specific antibody, 50 images (fields) were randomly acquired at 400x magnification using the software Image Pro version 4.5. After collecting the frames, positive cells were quantified in each of the 50 fields in every organ and the median of positive cell number was determined. All analyzes were accomplished in a blind test without prior knowledge of the studied groups. After quantification, frames exhibited in figures were selected as to be more informative according to specific areas in the analyzed tissues. S1 Table contains the raw data.

### In Situ Hybridization

The assessment of DENV in liver sections was performed by *in situ* hybridization using a digoxigenin-tagged probe (5’-TGACCATCATGGACCTCCA-3’) which anneals within the negative strand of the DENV RNA genome, as previously described [13]. Briefly, paraffin-embedded sections of tissues were deparaffinized and digested with pepsin (1.3 mg/ ml) for 4 min at room temperature. Tissues were incubated with the probe cocktail at 60°C for 5 min and then kept overnight at 37°C for denaturation and hybridization, respectively. Next, samples were washed with 0.2 x SSC and 2% bovine serum albumin at 55°C for 5 min. The probe-target complexes were revealed by the activity of alkaline phosphatase conjugated to anti-digoxigenin.

### Co-staining of DENV RNA and pro-inflammatory cytokines

Co-staining of virus and IFN*γ* or TNF*α* were performed, as previously described [13], using *in situ* hybridization and immunohistochemistry. The dengue case 2 was considered for this analysis. Briefly, the DENV probe was first tagged with 59 digoxigenin and locked nucleic acid (LNA) modified (Exiqon). Resulting complexes were visualized using an antidigoxigenin-alkaline phosphates conjugate and nitro-blue tetrazolium and 5-bromo-4-chloro-39-indolyphosphate as the chromogen. Detection of IFN*γ* or TNF*α* was then performed by immunohistochemistry (anti-IFN*γ* antibody—ABCAM ab133566-rabbit and anti-TNF*α*—ABCAM ab6671-rabbit) using Leica Bond Max automated platform (Leica Biosystems) and DAB as the chromogen. No counterstain was done. Data were analyzed by the computer based Nuance system (Caliper Life Sciences, Hopkinton, MA, USA) which separates the different chromogenic signals, converts them to fluorescent-based signals and combine them to determine co-expression.

### Statistical analyses

Data were analyzed with GraphPad prism software v5.1 (La Jolla, USA) using non-parametric statistical tests. Significant differences between analyzed groups (controls and DENV-patients) were determined using Mann-Whitney test with *p < 0.05.

## Results

### The liver as a target of immune-mediated mechanisms in dengue fatal cases

The liver is considered as an important target for DENV infection and is the most common organ to be involved in the disease. Hepatic alterations are key characteristics found in DENV cases. As observed in biopsies and autopsies of previously reported fatal cases [16], hepatocytes and Kupffer cells are described as important targets during DENV infection [17, 18]. For this reason, liver samples of the four DENV-3 fatal cases were first considered for our evaluations.

**Fig 1:**
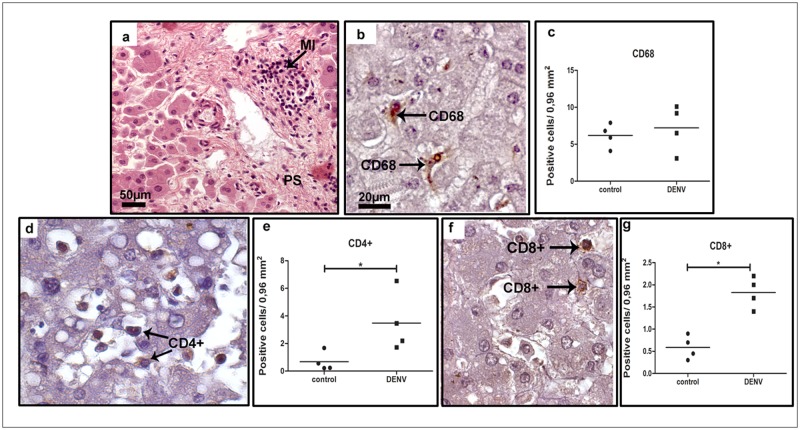
Characterization of cell subpopulations in liver tissues from DENV-3 fatal cases. Sections were stained with (a) H.E. or (b, d, f) incubated with specific antibodies in immunohistochemical assays. (a) Liver of a representative dengue case showing diffuse mononuclear infiltrates around the portal space; (b) detection of hyperplasic Kupffer cells (CD68^+^) observed mainly in sinusoidal capillaries of the dengue cases; (c) quantification of CD68^+^ cells in dengue cases and controls (non-dengue cases); (d) detection of CD4^+^ cells manly in portal space of the dengue cases; (e) quantification of CD4^+^ cells in controls and dengue cases; (f) CD8^+^ cells detected mainly in portal space; (g) quantification of CD8^+^ cells. MI—mononuclear cell infiltrates. Asterisks indicate differences that are statistically significant between dengue cases and control groups, (*p < 0.05). Staining controls are shown in S1 Fig.

Histopathological studies of all samples showed diffuse mononuclear cell infiltrates, mainly around the portal space (Fig 1 panel a). Detection of CD68^+^ cells revealed the presence of hyperplasic Kupffer cells and/or circulating macrophages (Fig 1 panel b), although quantification analysis did not show statistical difference when comparing dengue to control samples (Fig 1 panel c). Yet, we detected numerous CD4^+^ (Fig 1 panel d), and CD8^+^ (Fig 1 panel f) T cells placed mainly in sinusoidal capillaries. Quantification of these cell populations showed a significant increase in the number of T lymphocytes present in liver samples of dengue cases when compared to controls (about 5 and 3 fold, respectively—Fig 1 panels e and g).

**Fig 2:**
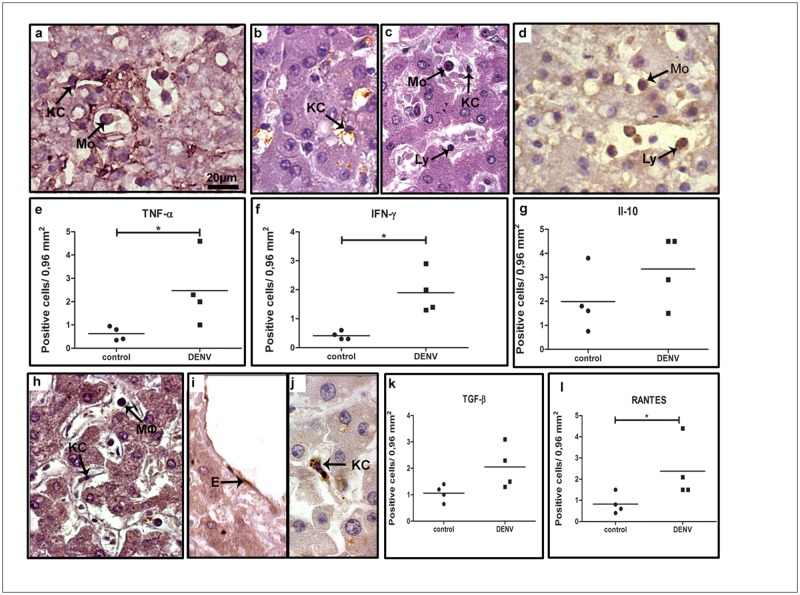
Detection of cytokine-producing cells in liver tissues. (a) Detection of TNF-*α* in macrophages and Kupffer cells in sinusoidal capillaries; (b and c) detection of INF-*α* in Kupffer cells and lymphocytes; (d) production of anti-inflammatory cytokines (IL-10 in monocytes and lymphocytes circulating in sinusoidal capillaries and TGF*β* detected in macrophages inside the portal space; (h) TGF-*β*-expressing macrophages and Kupffer cells in sinusoidal capillaries; (i and j) detection of RANTES in endothelium and Kupffer cells; (e, f, g, k and l) quantification of the number of cells expressing these cytokines in the hepatic tissue. Monocytes (Mo); Macrophages (M*ϕ*); Kupffer cells (KC); lymphocyte (Ly) and sinusoidal endothelium cells (E). Asterisks indicate differences that are statistically significant between groups (*p < 0.05). Staining controls are shown in S1 Fig.

In order to qualify the ongoing inflammatory process in the hepatic tissue, we also investigated the cytokine production by the mononuclear cell types found in the liver. In this case, cells expressing TNF-*α*, IFN-*γ*, IL-10, TGF-*β* and RANTES were considered for quantification. We observed a great number of cells producing these cytokines in the midzonal area and, to a lesser extent, in other hepatic areas. Production of TNF-*α* was detected by Kupffer cells and monocytes, mainly in the sinusoidal capillaries (Fig 2 panel a), while detection of IFN-*γ* was found mostly in lymphocytes, Kupffer cells and monocytes (Fig 2 panels b and c). In sinusoidal capillaries, we also detected groups of cells with an anti-inflammatory profile, such as IL-10-expressing monocytes and lymphocytes (Fig 2 panel d) and TGF-*β*-expressing macrophages and Kupffer cells (Fig 2 panel h). The chemokine RANTES/CCL5 was detected mainly in endothelium and Kupffer cells (Fig 2 panels i and j). Quantification of cells producing the inflammatory cytokines TNF-*α*, IFN-*γ* and RANTES/CCL5 revealed a significant increase (4-, 4.5- and 3-fold, respectively) in dengue group compared to control (Fig 2 panels e, f and l), while the number of cells expressing the anti-inflammatory cytokines IL-10 and TGF-*β* did not change significantly in either group (Fig 2 panels g and k).

### Histopathological analysis and cytokine profile present in the lungs of dengue fatal cases

Previous studies in our laboratory revealed that lung tissues from fatal dengue cases showed severe damages as represented by diffuse areas of hemorrhage and edema [13]. Here, we aimed to investigate a possible contribution of an exacerbated pro-inflammatory response that could be related to this local tissue impairment.

**Fig 3:**
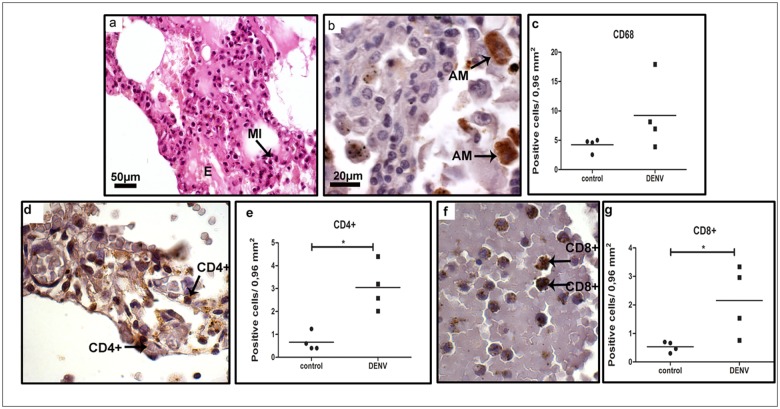
Characterization of mononuclear cell subpopulations in lung tissues collected from DENV-3 fatal cases. Lung tissue sections stained with (a) H.E. or (b, d and f) incubated with specific antibodies in immunohistochemistry assays. (a) Mononuclear infiltrates located in interstitial septa; (b) macrophages (CD68^+^ cells) observed in alveolar capillaries; (d and f) CD4^+^ and CD8^+^ T lymphocytes respectively, found in interstitial infiltrates septa; (c, e and g) quantification of cell subpopulations in the lung tissue. Macrophage (CD68); Mononuclear infiltrate (MI); edema (E); alveolar macrophage (AM); (CD4^+^) lymphocytes; (CD8^+^) lymphocytes. Asterisks indicate differences that are statistically significant between analyzed groups (*p < 0.05). Staining controls are shown in S2 Fig.

After analysis of lung sections of dengue cases we observed a diffuse mononuclear infiltrate in alveolar septa and edema areas (Fig 3 panel a), thus, indicating that this tissue could also be targeted by immune mechanisms triggered by infection. The immunohistochemical assay revealed the presence of macrophages (CD68^+^ cells) (Fig 3 panel b) in alveolar septa, CD4^+^ (Fig 3 panel d) and CD8^+^ T cells (Fig 3 panel f) mainly in the blood vessels. The quantification analysis revealed a 4-fold increase of lymphocytes in the dengue group when compared to non-dengue samples (Fig 3 panels e and g), whereas the number of CD68^+^ was not statistically different from the controls (Fig 3 panel c).

**Fig 4:**
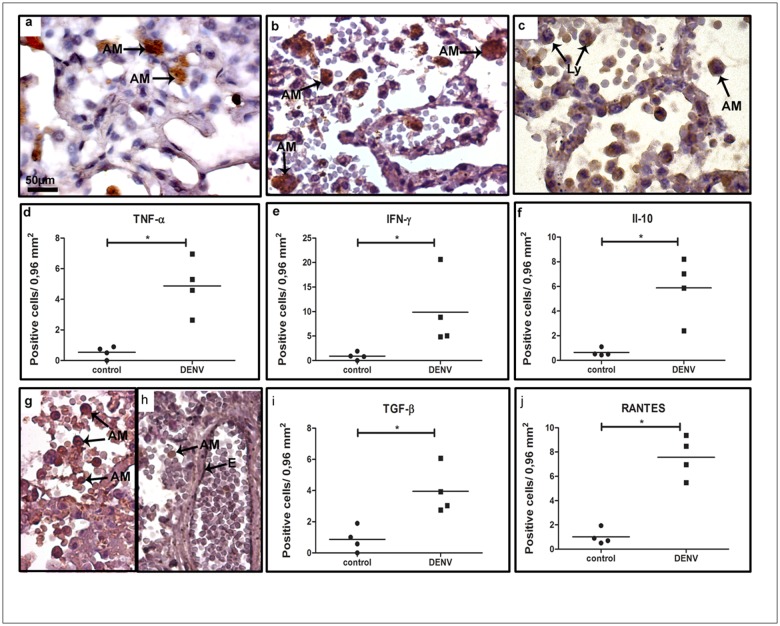
Cytokine-producing cells profile in lung tissues of dengue fatal cases. Immunohistochemical analysis of lung sections collected from dengue fatal cases exhibited (a) TNF-*α* and (b) INF-*γ* production in alveolar macrophages; (c) IL-10 detected in monocytes/macrophages and lymphocytes in alveolar septa; (g) TGF-*β* produced by numerous macrophages and lymphocytes in the septa; (h) RANTES detected in endothelium cells and several macrophages in pulmonary tissue; (d-f, i and j) quantification of cytokine-expressing cells in the lung sections. Monocyte (Mo); Alveolar macrophage (AM); Endothelium (E); Lymphocytes (Ly). Asterisks indicate differences that are statistically significant between dengue cases and controls (*p < 0.05). Staining controls are shown in S2 Fig.

We next aimed to identify the local cytokine profile in the lungs to characterize the ongoing inflammatory process in fatal cases of severe dengue. The evaluation of cytokine-expressing cells in the tissues of the dengue group exhibited pro- and anti-inflammatory profiles occurring simultaneously, what revealed an atypical elicited immunity. TNF-*α*, which is an important cytokine related to dengue pathogenesis, was detected mainly in alveolar macrophages. (Fig 4 panel a). Increased numbers of TNF-*α*- (Fig 4 panel d), IFN-*γ*- (Fig 4 panels b and e), IL-10- (Fig 4 panels c and f) and TGF-*β*- (Fig 4 panels g and i) expressing cells such as macrophages and lymphocytes were characterized in the tissues of dengue cases, when compared to controls. Vascular permeability impairment was also addressed in dengue cases by the presence of several RANTES-expressing endothelial cells and alveolar macrophages in the perivascular space (Fig 4 panel h). The quantification of these subpopulations was found to be increased in dengue cases when compared to controls (Fig 4 panel j).

### Kidneys in severe dengue are targeted by pro-inflammatory cells

Kidney involvement with dengue virus infection is being recognized, since the prevalence of proteinuria and hematuria has been reported as high as 70-80% [19]. Mechanisms that may drive kidney complications in dengue are not clear but possibly result from indirect pathways via host immunity. Due to these knowledge gaps, renal sections extracted from post-mortem dengue cases were also considered for investigation.

**Fig 5:**
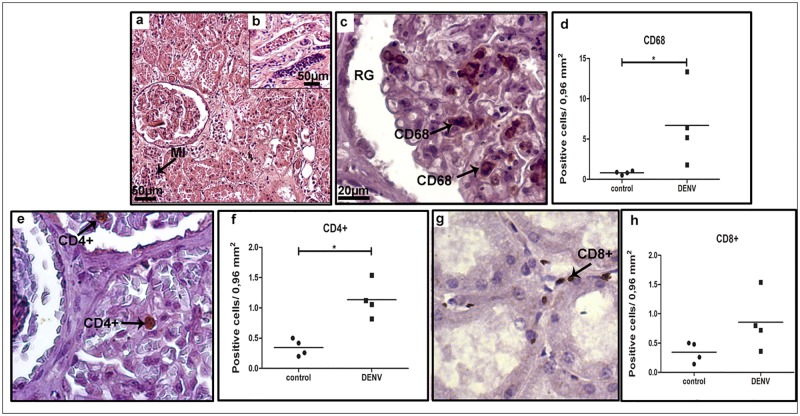
Characterization of mononuclear cells found in renal sections of post-mortem dengue cases. (a and b) A representative kidney section stained with H.E. showing diffuse mononuclear cell infiltrate; (c) detection of (CD68^+^) in mesangial cells in glomeruli; (e and g) circulating CD4^+^ lymphocytes detected inside renal glomeruli and CD8^+^ cells observed in the interstitial space, respectively; (d, f and h) quantification of cell subpopulations in the kidney tissue. Mesangial cells (CD68); (CD4^+^) lymphocytes; (CD8^+^) lymphocytes. RG—renal gromerulus. Asterisks indicate differences that are statistically significant between groups (*p < 0.05). Staining controls are shown in S3 Fig.

Tissue analysis of severe cases revealed a diffuse mononuclear infiltrate, more pronounced in cortical and medullar regions (Fig 5 panels a and b, respectively). CD68^+^ cells were detected mainly in mesangial cells (monocyte or smooth muscle origin, responsible for filtration, structural support, and phagocytosis), located in the glomerulus (Fig 5 panel c). The quantification of CD68^+^ cells revealed an 8-fold increment of this population in dengue cases when compared to non-dengue patients (Fig 5 panel d). The number of CD4^+^ T cell lymphocytes, located primarily within the renal glomerulus, was also found to be increased in dengue group renal sections, when compared to controls (Fig 5 panels e and f). CD8^+^ T cells were detected mainly in the medullar zone (Fig 5 panel g) and their quantification showed no statistical difference between dengue and control groups (Fig 5 panel h).

**Fig 6:**
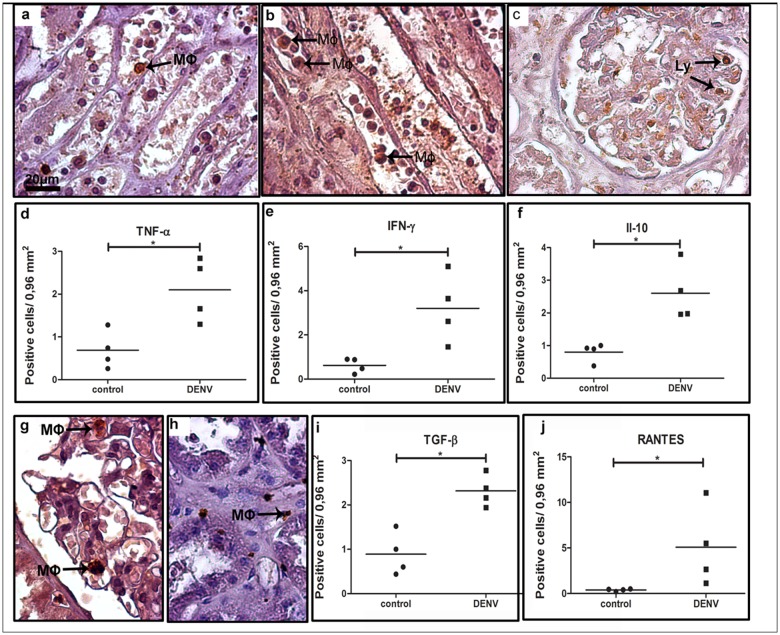
Cytokine-producing cells in renal tissues of dengue cases. (a) TNF-*α* detected in monocytes located in blood vessels; (b) Production of INF-*γ* observed in circulating macrophages and lymphocytes located in the interstitial space; (c) IL-10 found in endothelial cells of glomerulus; (g) TGF-*β* production by lymphocytes present mainly inside renal glomeruli; (h) RANTES detected in macrophages and lymphocytes in the interstitial renal space; (d-f, i and j) quantification of the number of cells expressing the above cytokines. Macrophages (M*ϕ*); Lymphocytes (Ly). Asterisks indicate differences that are statistically significant between analyzed groups (*p < 0.05). Staining controls are shown in S3 Fig.

The evaluation of cytokine profile in renal samples revealed TNF-*α* being produced mainly by monocytes/macrophages present in the medullar region (Fig 6 panel a). IFN-*γ*-producing cells, such as macrophages, were found in blood vessels located also in the medullar region (Fig 6 panel b). The anti-inflammatory cytokines IL-10 and TGF-*γ* were observed mostly in lymphocytes within the renal glomerulus (Fig 6 panel c) and macrophages (Fig 6 panel g), while RANTES production was detected mainly in macrophages only (Fig 6 panel h). Quantification of cells producing these cytokines revealed a general increase in dengue cases when compared to controls. The number of cells producing of TNF-*α* and IFN-*γ* in dengue cases was 3- and 5-fold higher, respectively, after comparison with non-dengue cases (Fig 6 panels d and e). Concerning the number of cells expressing TGF-*β* (Fig 6 panel i), IL-10 (Fig 6 panel f) and RANTES (Fig 6 panel j), in dengue cases we noted increments of about 2.5-, 3- and 13-fold, respectively.

### DENV-specific induction of pro-inflammatory response

In order to confirm the participation of DENV-infected cells in inducing the local pro-inflammatory response, DENV RNA and cytokine production were co-tested in host samples. For this evaluation, the liver was elected due to its importance as a target organ in dengue pathogenesis.

**Fig 7:**
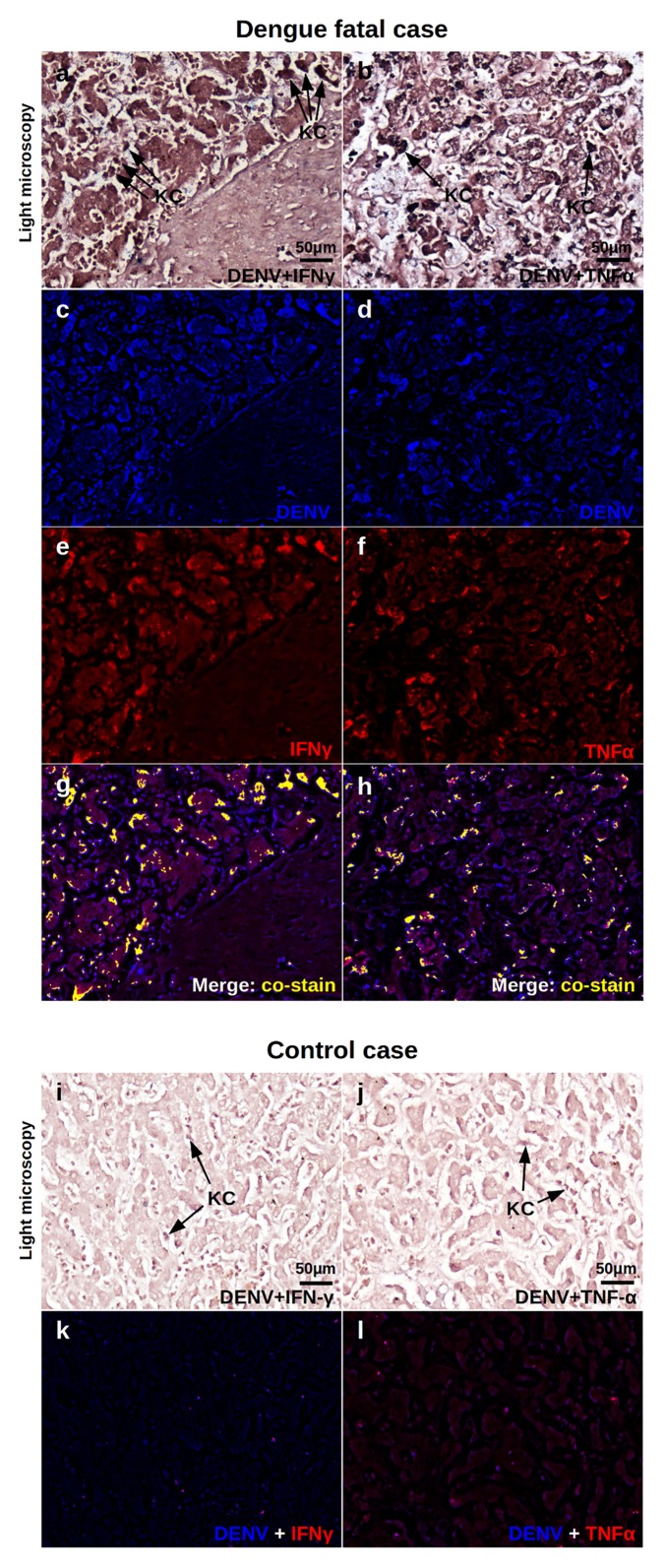
Co-expression of DENV and pro-inflammatory cytokines in the liver. Liver samples of dengue fatal case 2 were processed for *in situ* hybridization and IHC procedures. DENV was detected by a probe that aneals to a conserved sequence within the viral RNA negative strand. IFN-*γ* and TNF-*α* were assessed by immunohistochemistry assay. Probe-target complexes were revealed by alkaline phosphatase activity and cytokines were identified by standard DAB reactions. The chromogenic signals were converted to fluorescent-based signals using a computer based Nuance system. Representative images are shown: (a, b) Light microscopy of sinusiodal areas of dengue case stained for DENV RNA and IFN-*γ* or TNF-*α*, respectively. (c, d) Expression of DENV RNA (blue) in samples stained with IFN-*γ* or TNF-*α*, respectively. (e, f) Expression of IFN-*γ* or TNF-*α* (red) in in samples stained with DENV RNA, respectively. (g, h) Merged signals of DENV RNA and IFN-*γ* or TNF-*α* where co-expression is exhibited in yellow, respectively. (i, j) Light microscopy of sinusiodal areas of a non-dengue case stained for DENV RNA and IFN-*γ* or TNF-*α*, respectively. (k, l) Comtrol samples showing DENV and IFN-*γ* or TNF-*α* fluorescent signals, respectively. KC—Kupffer cells.

As expected, the light microscopy of a dengue fatal case exhibited mononuclear cell infiltrates with the presence of Kupffer cells (KC) in the sinusoid capillaries (Fig 7 panels a and b), while non-dengue control presented regular hepatic structures with resident KCs (Fig 7 panels i and j). As detected by *in situ* hybridization and IHC, the dengue case showed many areas of co-expression in the hepatic tissue considering DENV RNA and IFN-*γ* or TNF-*α* (Fig 7 panels g and h). In this case, Kupffer cells were the main targets of co-staining. The control case presented no staining for either DENV or the studied cytokines.

These data confirmed, under a qualitative basis, the specific DENV-induction over pro-inflammatory cytokine response and also addressed the virus spread versus IFN-*γ* or TNF-*α* expression in the liver sample of a dengue fatal case.

## Discussion

The elucidation of mechanisms that underlie the development of severe dengue is still seen as a major challenge in the field [20]. The progress of dengue research concerning such aspects has been hampered by a set of factors that include the peculiar nature of DENV infection together with the absence of an immunocompetent animal approach capable of mimicking severe dengue symptoms [21]. Under this scenario, the investigation of post-mortem samples extracted from severe dengue human cases would bring valuable information to better understand the pathogenic base of the disease. Immune mechanisms are thought to drive the mild flu-like illness (DF) to the severe hemorrhagic stages of dengue, since such manifestations occur after virus clearance from the circulation [22]. In this work, four fatal human cases that experienced the severe hemorrhagic symptoms of dengue had three of their peripheral sites (liver, lung and kidney) investigated. Organs were elected according to previous known relevance or knowledge gaps related to dengue [16–19, 23]. Research was focused on the cellular immunity, in which mononuclear cell migration and cytokine production were considered to the evaluation of host response by the fatal cases of severe dengue. In a previous report from our laboratory, we found that the same post-mortem samples presented tissue damages that were consistent with severe dengue clinical cases [13]. The inspection of these tissues under an immunological approach was our major concern in this work, since evidences of an exacerbated host immunity would somehow be linked to organ impairments. From a first panoramic view, all evaluated sites from fatal cases (liver, lungs and kidneys) were targeted by mononuclear cell migration, such as macrophages and T cells. Apart from this, the analyzed peripheral organs exhibited higher levels of pro-inflammatory cells, as found by their production of TNF-*α*, IFN-*γ* and RANTES. Those observations strongly supported the proposed theories claiming that exaggerated [24, 25] or misdirected [8, 26–28] T-cell responses would eventually lead the host to severe clinical stages.

Numerous reports describe the release of different cytokines and soluble receptors during dengue infection [25, 29, 30], which has also been associated to the unfavorable disease outcome [31]. In this work, increased levels of RANTES-producing endothelial cells may have contributed to the occurrence of cell infiltrates, since this chemokine signals for cell movement from the bloodstream into tissues [32, 33]. Therefore, that would infer a close connection between RANTES and the altered vascular permeability events related to severe dengue. In this case, higher RANTES production and secretion would favor plasma leakage and lymphocyte cell infiltration into the liver, lungs and kidneys, hence, potentially mediating the inflammatory response found in these peripheral organs. Among all chemokines, RANTES is particularly associated with viral infections [34]. RANTES, also known as CCL5, is an early expressed chemokine induced by pattern recognition receptors, but can also be induced by TNF-*α* and IFN-*γ* at late stages of infection [35], that is the case of a terminal dengue situation. The discussion about chemokine signaling and its effects over infectious diseases can sometimes be controversial in the literature. Together with other chemotactic effectors (CXCL9/10/11) and immune cell response-modulating cytokines (IL-6, IL-7 and BAFF), RANTES has been associated with immune enhancement and increased vascular permeability following dengue virus infection [36, 37]. Conversely, *in vitro* experiments revealed that hantavirus can infect human lung microvascular endothelial cells (HMVEC-Ls) and stimulate secretion of RANTES by these cells without increasing vascular permeability [38]. Together, these observations still arise skeptical thoughts that lead to a more careful discussion concerning a direct link between RANTES and vascular permeability enhancement in dengue.

The higher amounts of TNF-*α*-producing cells characterized in the post-mortem samples were in line with reports in the literature. TNF-*α* is considered as a major pro-inflammatory mediator in dengue infections since its activity has been linked to the immunopathogenesis of the disease [28, 39]. Apart from the existing drawbacks regarding animal models to reproducing dengue, reports showed that the inhibition of TNF-*α* by administrating specific antibodies was associated with reduced severity [39, 40]. Although the TNF-*α* inhibition assay was key to infer its importance in severe dengue, in practical terms, targeting TNF with antibody or receptor antagonists for treating human diseases is controversial. In other diseases with immunological basis, not all patients were helped despite the clinical effectiveness of anti-TNF aproaches. This fact, perhaps, reflected the existence of distinct underlying mechanisms that drive the symptoms apart from the TNF network [41].

Along with TNF-*α*, IFN-*γ* was also found to be increased in terms of production by infiltrated mononuclear cells in peripheral tissues of the studied fatal dengue cases. Hence, the present work showed INF-*γ* as a pro-inflammatory element that may contribute to tissue distress, also representing an *in situ* evidence of disease severity. In a recent report, metabolomics was adopted to screen dengue-induced metabolites in 116 dengue patients (60 presenting DF and 56 with severe dengue). It was found that circulating IFN-*γ* combined with serotonin levels provided accurate early prognosis of severe dengue, thus revealing its importance and an additional clinical usage of this pro-inflammatory cytokine to assess severity [42].

The mononuclear cell migration that targeted the studied tissues were also proposed to be correlated with local impairments. In liver samples, CD4^+^ T cells, Kupffer cells and monocytes were characterized near hepatocellular necrosis and steatosis in the presence of pro-inflammatory cytokines, IFN-*γ* and TNF-*α*. Additional alterations on hepatocytes, such as nuclear vacuolar degeneration and the presence of swollen mitochondria, based on preceeding studies, suggested an ongoing mechanism of apoptotic cell death possibly mediated by the cytokine environment [13, 43–47]. In our previous report, the lung scenario of the studied dengue fatal cases was marked by a peculiar histopathological evidence. The presence of septum thickening with an increase of cellularity characterized a hyaline membrane formation possibly due to dengue shock syndrome [13]. As the activity of pro-inflammatory cytokines (IFN-*γ* and TNF-*α*) were previously associated with lung injury [48, 49], we envisioned a possible correlation between local cytokine production and tissue alterations. Under this idea, the hyaline membrane structure could be formed in function of an exacerbated cytokine release by hyperplasic alveolar macrophages combined with tissue alterations such as edema and hemorrhage, which is also found in other non-related diseases [50, 51]. Parenchyma and circulatory damages found in kidney samples would likely be immune-mediated due to the presence of mononuclear infiltrates in cortical and medullar regions. A recent report correlated kidney injuries of severe dengue cases with the local recruitment of T cells [52]. In line with our findings, authors also found that infiltrated CD8^+^ T lymphocytes were outnumbered by CD4^+^ T cells, suggesting an important role of this subpopulation in tissue damages. Hence, considering all analyzed tissues under the above circumstances, it would be reasonable to suggest a key role of cellular immunity to determine local tissue alterations/dysfunctions. It is important to note that when referring to CD8-expressing lymphocytes a number of different cell populations, other than classical T cells, may also be taken into account. Due to the close morphology, NK cells and mucosal-associated invariant T (MAIT) cells are examples of CD8-expressing subsets that may also present roles in severe dengue. While the participation of NK cells in DENV infection has been extensively reported [53–57], the role of MAIT cells in such disease is still a novel and eye-catching mater of debate [58].

At this point, a relevant question emerges considering the induction of the evident immune reaction found in the analyzed tissues. Considering the environmental circumstances and the debilitating situation of a patient under severe dengue, it would be possible that immunity induced against other opportunistic pathogens would also be occurring. Such hypothesis can not be excluded, however, the DENV-specific participation over the elicited immunity was confirmed in this work. *In situ* hybridization together with IHC experiments showed, qualitatively, that DENV produces a direct effect over pro-inflammatory cytokine production. As noted by the historic of symptoms, laboratory workup [13] and our present evaluation, we consider that the major element leading to the observed effects over immunity is the infection by DENV.

Under the immune-mediated theory for the pathogenesis of dengue, anti-inflammatory or regulatory cytokines would, at first glance, contribute to a better prognosis. An interesting fact that occurred during tissue analysis (mainly liver and kidney samples) was the detection of anti-inflammatory cells with production of IL-10 or TGF-*β*, even in the presence of the above discussed pro-inflammatory environment. One hypothesis to explain this atypical finding would be the triggering of a host immunity attempt to circumvent the local inflammatory process. Under this idea, as the studied tissues were previously characterized with local impairments [13], this would indicate the existence of a strong ongoing inflammatory effect capable of overwhelming regulatory responses. In line with this assumption, a report in the literature showed that the overexpression of pro-inflammatory cytokines has been suggested to cause an inhibitory influence on regulatory cells [59]. Another recent report revealed that, in fact, a disturbance in the balance between inflammatory (IL-6 and IL-8) and anti-inflammatory (IL-10) cytokines, would characterize possible mechanisms related to the occurrence of hemorrhagic manifestations in dengue [60]. Additionally, IL-10 has also been interpreted as a marker of disease progression in severe dengue cases [61]. Regardless of these corroborating evidences from the literature, we still find it difficult and risky to draw conclusions over these aspects from the studied post-mortem samples. Investigations considering the time dependency of the immune events along with the host clinical evolution would still be necessary for a more consistent description about this pro- versus anti-inflammatory balance.

In conclusion, the study of post-mortem samples from peripheral organs of severe dengue cases provided valuable information about the local environment under an immunological approach. The existence of a strong ongoing pro-inflammatory response was suggested to be occurring in liver and manly in lung and kidney samples. The presence of mononuclear cell infiltrates, higher counts of pro-inflammatory cells (as found by the production of TNF-*α*, IFN-*γ* and RANTES) and the apparent outrun of inflammation over anti-inflammatory elements (such as IL-10 and TGF-*β*) were the major evidences for such characterization. Apart from many other known hypotheses for the determination of severe dengue cases [20], this work provided additional evidences that supported the cellular immune-mediated theories, hence, contributing to a better understanding of dengue pathogenesis.

## Supporting Information

S1 Fig Liver tissue controls of immunohistochemistry assays. Histological sections of a non-dengue case organ showing regular structures and preserved parenchyma. Slides were stained with anti-CD68 (a), anti-CD4 (b), anti-CD8 (c), anti-TNF*α* (d), anti-IFN*γ* (e), anti-IL-10 (f), anti-TGF*β* (g) and anti-RANTES (h).
(TIF)

S2 Fig Lung tissue controls of immunohistochemistry assays. Histological sections of a non-dengue case organ showing regular structures and preserved parenchyma. Slides were stained with anti-CD68 (a), anti-CD4 (b), anti-CD8 (c), anti-TNF*α* (d), anti-IFN*γ* (e), anti-IL-10 (f), anti-TGF*β* (g) and anti-RANTES (h).
(TIF)

S3 Fig Kidney tissue controls of immunohistochemistry assays. Histological sections of a non-dengue case organ showing regular structures and preserved parenchyma. Slides were stained with anti-CD68 (a), anti-CD4 (b), anti-CD8 (c), anti-TNF*α* (d), anti-IFN*γ* (e), anti-IL-10 (f), anti-TGF*β* (g) and anti-RANTES (h).
(TIF)

S1 Table Quantification of positive cells by immunohistochemistry. (XLSX)
