# Correction: Peripheral Organs of Dengue Fatal Cases Present Strong Pro-Inflammatory Response with Participation of IFN-Gamma-, TNF-Alpha- and RANTES-Producing Cells

**DOI:** 10.1371/journal.pone.0195140

**Published:** 2018-03-26

**Authors:** Tiago F. Póvoa, Edson R. A. Oliveira, Carlos. A. Basílio-de-Oliveira, Gerard J. Nuovo, Vera L. A. Chagas, Natália G. Salomão, Ada Maria de Barcelos Alves, Ester M. Mota, Marciano V. Paes

Dr. Ada Maria de Barcelos Alves should be included in the author byline instead of the Acknowledgments. She should be listed as the seventh author and affiliated with Laboratory of Biotechnology and Physiology of Viral Infections, Oswaldo Cruz Institute, Oswaldo Cruz Foundation, Rio de Janeiro, Brazil. The contributions of this author are as follows: Conceived and designed the experiments, performed the experiments, analyzed the data, and contributed reagents/materials/analysis tools.
