# Supplementary figures and images for: Peripheral Organs of Dengue Fatal Cases Present Strong Pro-Inflammatory Response with Participation of IFN-Gamma-, TNF-Alpha- and RANTES-Producing Cells
Source: PLoS One. 2016 Dec 22;11(12):e0168973. doi: 10.1371/journal.pone.0168973 (PMC5179082; doi:10.1371/journal.pone.0168973)

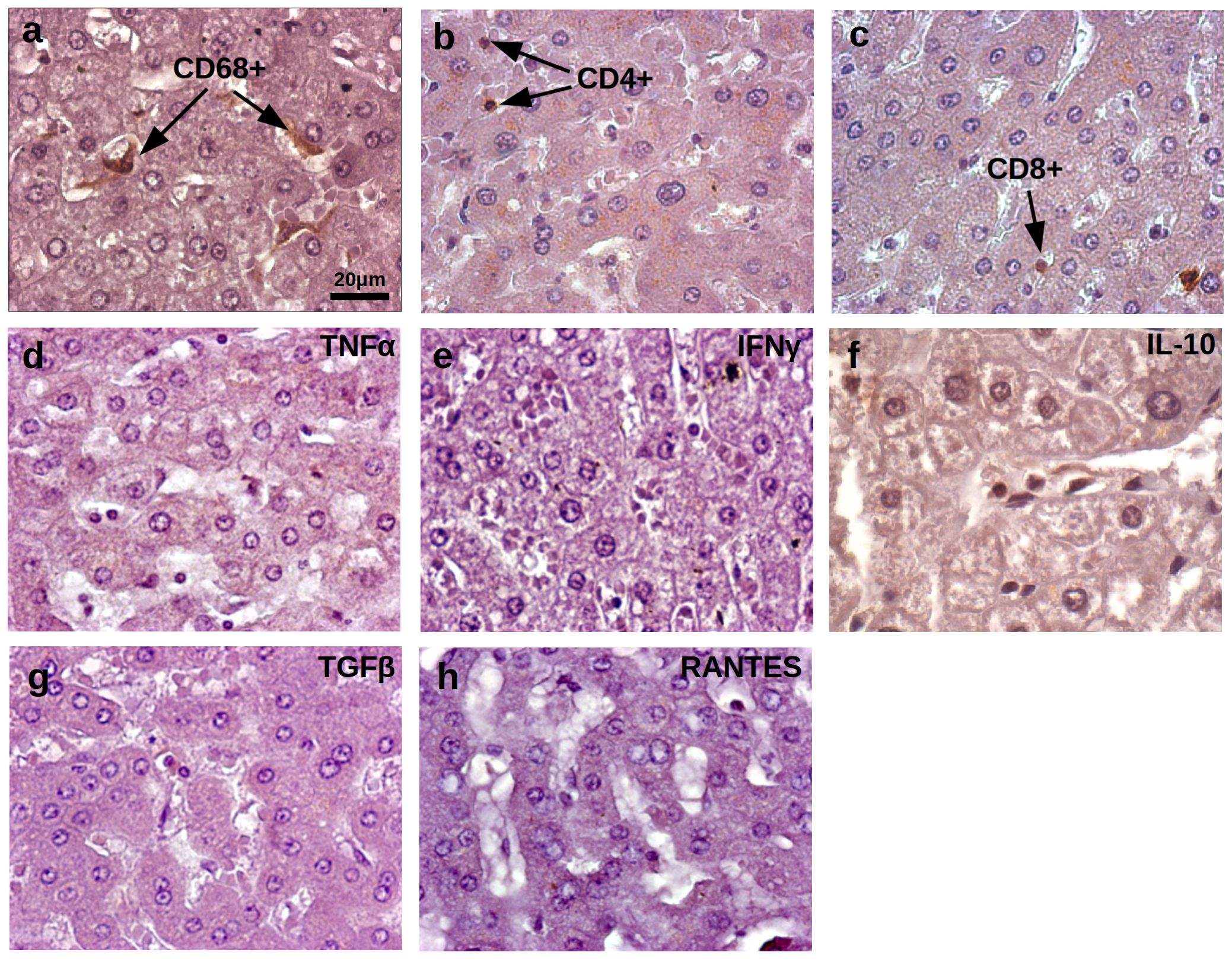

Supplement: S1 Fig — Histological sections of a non-dengue case organ showing regular structures and preserved parenchyma. Slides were stained with anti-CD68 (a), anti-CD4 (b), anti-CD8 (c), anti-TNFα (d), anti-IFNγ (e), anti-IL-10 (f), anti-TGFβ (g) and anti-RANTES (h). (TIF) [file pone.0168973.s001.tif]

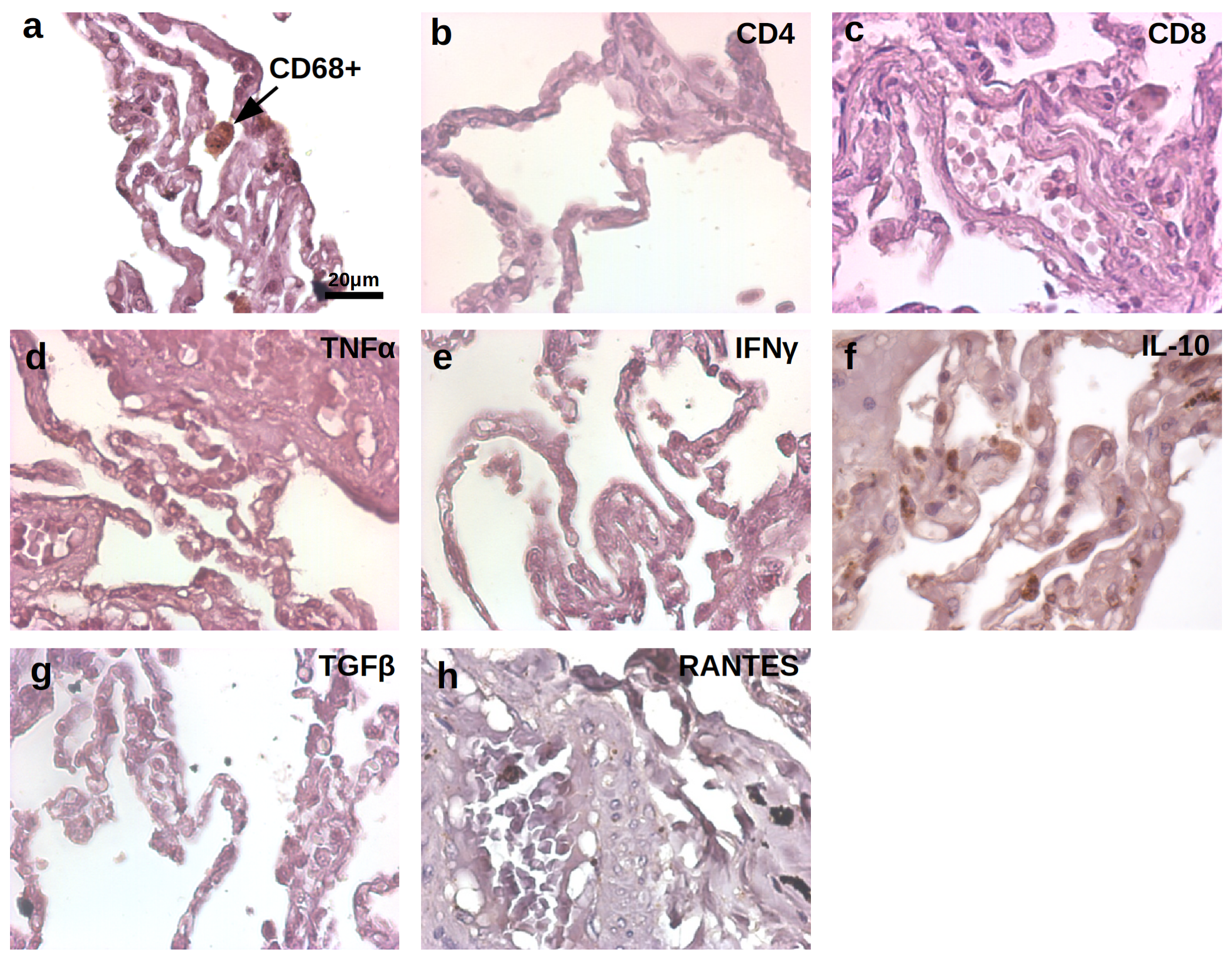

Supplement: S2 Fig — Histological sections of a non-dengue case organ showing regular structures and preserved parenchyma. Slides were stained with anti-CD68 (a), anti-CD4 (b), anti-CD8 (c), anti-TNFα (d), anti-IFNγ (e), anti-IL-10 (f), anti-TGFβ (g) and anti-RANTES (h). (TIF) [file pone.0168973.s002.tif]

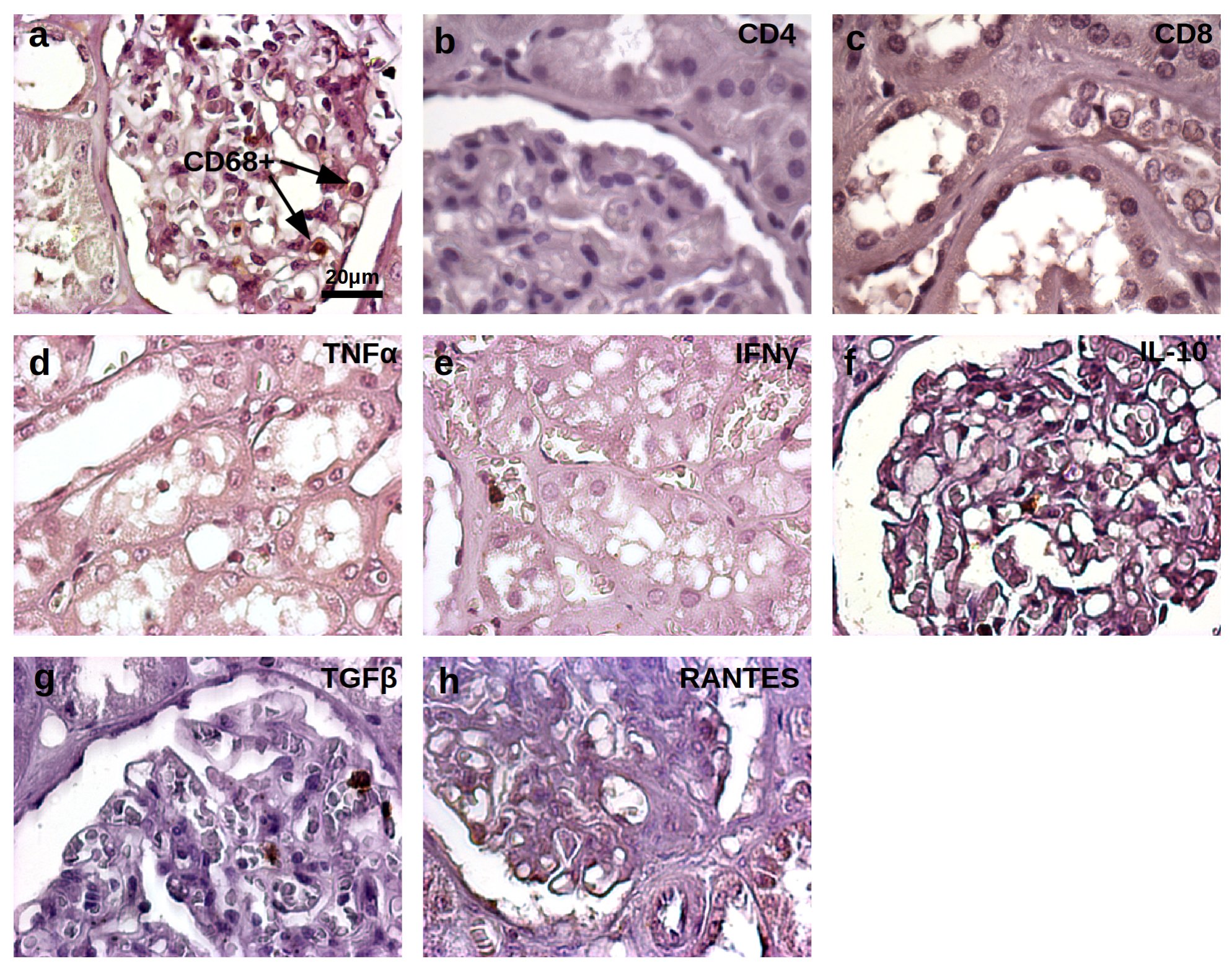

Supplement: S3 Fig — Histological sections of a non-dengue case organ showing regular structures and preserved parenchyma. Slides were stained with anti-CD68 (a), anti-CD4 (b), anti-CD8 (c), anti-TNFα (d), anti-IFNγ (e), anti-IL-10 (f), anti-TGFβ (g) and anti-RANTES (h). (TIF) [file pone.0168973.s003.tif]
